# Importance of two-dimensional cation clusters induced by protein folding in intrinsic intracellular membrane permeability†

**DOI:** 10.1039/d2cb00098a

**Published:** 2022-07-13

**Authors:** Shigeru Negi, Mami Hamori, Yuka Kawahara-Nakagawa, Miki Imanishi, Miku Kurehara, Chieri Kitada, Yuri Kawahito, Kanae Kishi, Takayuki Manabe, Nobuyuki Kawamura, Hiroaki Kitagishi, Masato Mashimo, Nobuhito Shibata, Yukio Sugiura

**Affiliations:** Faculty of Pharmaceutical Science, Doshisha Women's University, Koudo Kyotanabe Kyoto 610-0395 Japan snegi@dwc.doshisha.ac.jp; Graduate School of Life Science, University of Hyogo 3-2-1 Kouto, Kamigori-cho Ako-gun Hyogo 678-1297 Japan; Institute for Chemical Research, Kyoto University Uji Kyoto 611-0011 Japan; Graduate School of Biomedical and Health Sciences, Hiroshima University 1-2-3 Kasumi Minami-ku Hiroshima 734-8553 Japan; Clinical Research Support Center, Asahikawa Medical University Hospital 2-1-1-1 Midorigaokahigashi Asahikawa 078-8510 Japan; Education Center for Pharmacy, Faculty of Pharmaceutical Sciences, Niigata University of Pharmacy and Applied Life Sciences 265-1 Higashijima, Akiha-ku Niigata City Niigata 956-8603 Japan; Department of Molecular Chemistry and Biochemistry, Faculty of Science and Engineering, Doshisha University Kyotanabe Kyoto 610-0321 Japan

## Abstract

We investigated the cell penetration of Sp1 zinc finger proteins (Sp1 ZF) and the mechanism *via* which the total cationic charge and distribution of cationic residues on the protein surface affect intracellular trafficking. Sp1 ZFs showed intrinsic cell membrane permeability. The intracellular transfer of Sp1 ZFs other than 1F3 was dependent on the total cationic charge. Investigation of the effect of cationic residue distribution on intracellular membrane permeability revealed that the cellular uptake of unfolded Zn^2+^-non-coordinating Ala mutants was lower than that of the wild type. Therefore, the total cationic charge and distribution of cationic residues on the protein played crucial roles in intracellular translocation. Mutational studies revealed that the two-dimensional cation cluster on the protein surface significantly improved their cellular uptake. This study will contribute to the design of artificial cargoes that can efficiently transport target substances into cells.

## Abstract 2

Two-dimensional cation clusters formed on the surface of proteins play an important role in their intracellular translocation.

## Graphical abstract

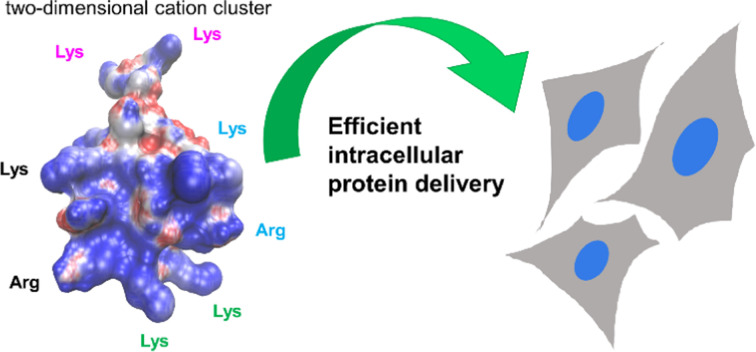

## Introduction

Biopolymers play important roles as materials for biomedicines such as protein and nucleic acid drugs.^1–6^ Currently, proteins are used as practical therapeutic agents. Most target sites of protein drugs developed so far, including antibody drugs, have an extracellular location.^7–9^ This is because the perturbation of intracellular targets with protein drugs is still challenging. Thus, the total cationic charge could significantly increase for more efficient intracellular delivery. Exogenous proteins were required in the few successful cases,^10^ as the high hydrophobicity of the cell membrane is a formidable barrier to the entry of biopolymers, including highly polar protein drugs, into the cell.^11,12^ Therefore, the development of effective and practical intracellular delivery technologies for protein drugs is crucial.^13–15^

Various reagents have been developed to introduce proteins into mammalian cells, including lipid-bound compounds, nanoparticles, polymers, protein-based containers, virus-like particles, and exosomes.^16–28^ Furthermore, researchers have used cell-permeable peptides (CPPs) with protein drugs for penetrating the cell membrane.^29–32^ The most common CPPs are HIV-1 transcription factor (Tat) peptide, oligoarginine, and penetratin from *Drosophila antennapedia*.^33–40^

Attempts are being made to introduce CPPs into cells by fusing them with target proteins, allowing their use as protein transduction domains (PTDs). This approach can be practically used for the intracellular delivery of various compounds, including biopharmaceuticals. However, some CPPs are cytotoxic because of their highly cationic nature.^41,42^ Therefore, drug delivery vectors that are safe and effective for cells *in vivo* are being developed. Furthermore, developing a powerful protein delivery platform will expand the application of protein-based research reagents and therapeutics.

Liu *et al.* developed a “supercharged” green fluorescent protein (GFP) by extensively mutating the amino acid residues exposed on the protein surface of GFP and examined its membrane permeability and ability to introduce other proteins into cells.^43,44^ GFP variants have a high theoretical net charge of −30 to +48. Positively supercharged GFPs can bind to cell surface proteoglycans, undergo endocytosis in an energy- and clathrin-independent manner, and efficiently invade multiple mammalian cells. Furthermore, super-positive GFP can be used to introduce siRNAs or plasmid DNA into cell lines without eliciting any cytotoxic effect, thereby allowing for genome editing and making this GFP variant a powerful and versatile biopharmaceutical platform.^45,46^ Moreover, the supercharged proteins have been found to possess better cell membrane permeability than conventional CPPs because of their high positive charge.^43,44^ Thus, the total cationic charge could be significantly increased for more efficient intracellular delivery. Similarly, Schepartz *et al.* introduced a cationic amino acid into the helical structure of type II polyproline to create an artificial peptide with a high total cationic charge while maintaining the structure. The authors showed that peptides with enhanced cationic charge exhibited higher cell membrane permeability than conventional CPPs and that an increase in the total amount of positive charge enhanced cellular uptake.^47,48^

In addition, some naturally occurring proteins with extremely high positive charges can deliver proteins into cells in a functional manner.^49^ One example is the DNA binding zinc finger (ZF) transcription factor, which is the target of this study. Schepartz *et al.* were the first to investigate cell membrane permeability using the ZF motif, ZF5.3, and the underlying mechanism by varying the number of Arg residues in the primary sequence.^50^ Several groups have also studied the cell membrane-specific permeability of ZFs and their application for the intracellular delivery of functional molecules.^51–57^ Thus, the ability to permeate the cell membrane may be a part of the intrinsic biological function of some naturally occurring proteins, which may contribute to hitherto unknown biological roles *in vivo*.

Therefore, the efficient intracellular delivery of proteins can be achieved using natural proteins as templates and redesigning them for a higher total cationic charge on the surface while retaining their folding structure. In short, we speculated that the efficient formation of two-dimensional cation clusters on the protein surface platform due to folding, rather than simply increasing the overall cationic charge of the peptide or protein, would be a crucial factor for efficient protein transport into the cell. We thus investigate the mechanism *via* which two-dimensional cation clusters on a protein surface affect cellular uptake by controlling the folding of a protein with a fixed total charge instead of changing the total positive charge of the protein *via* mutations. ZFs are one of the best model proteins for this purpose. ZF induces the formation of a ββα structure, which is essential for DNA binding ability, by coordinating with Zn^2+^.^58,59^ The mutation of Cys and His, which are involved in coordination, can control Zn^2+^ binding and consequently the formation of the ββα structure. The regulation of the ZF structure by Zn^2+^ coordination, combined with its inherent membrane permeability, can be used to investigate the effect of the two-dimensional cation clusters formed on the protein surface on cell membrane permeability.

**Fig. 1:**
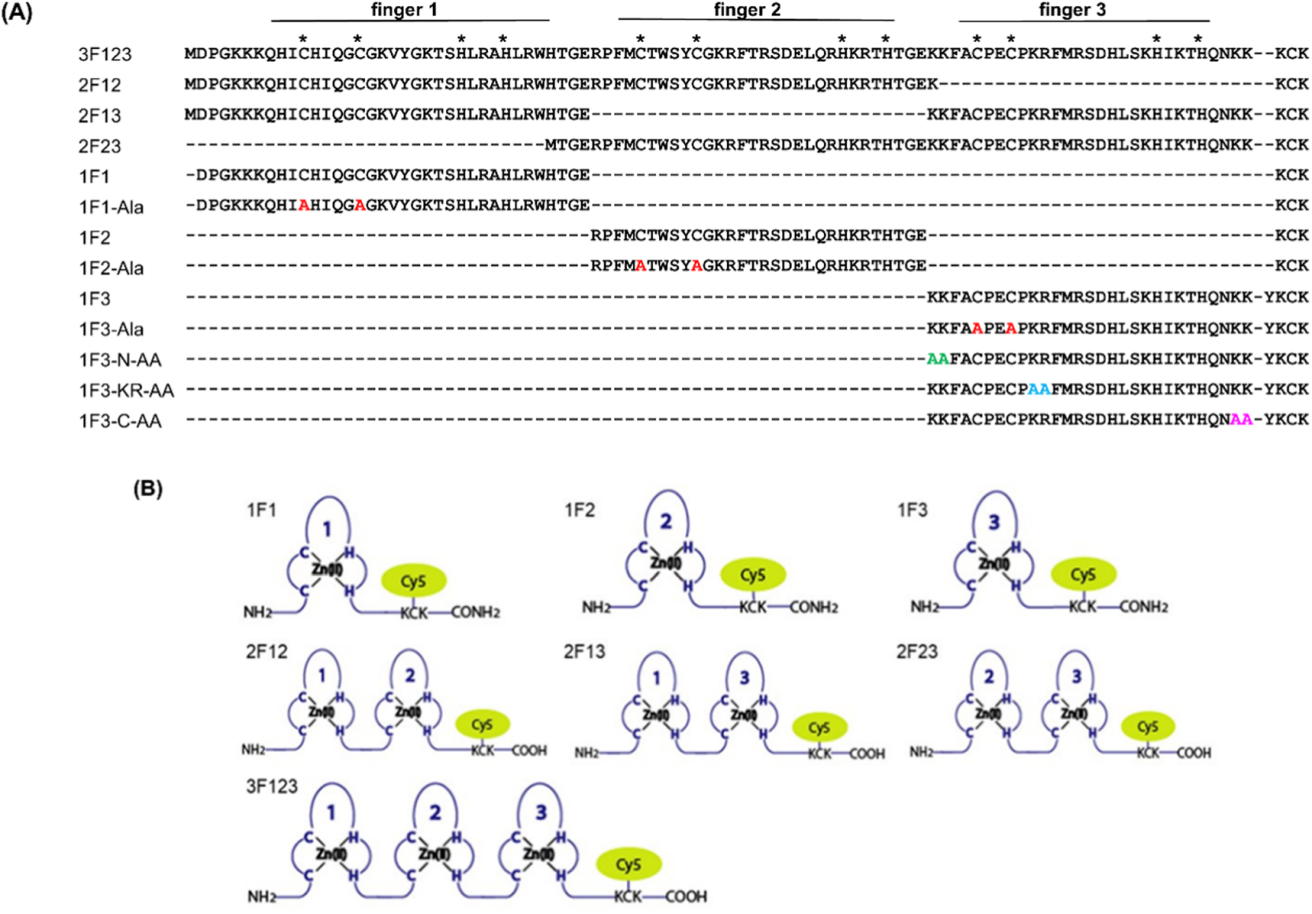
(A) Amino acid sequences of Sp1 ZFs. Cys and His residues involved in Zn^2+^ coordination are marked with asterisks. Mutation sites in the 1F1-Ala, 1F2-Ala, and 1F3-Ala sequences are shown in red. The Ala substitution sites in the 1F3-N-AA, KR-AA, and C-AA sequences are represented in green, light blue, and pink, respectively. (B) Representation of Cy5-labeled ZF peptide based on Sp1 ZF.

Previously, we have examined the membrane permeability of full-length and deficient mutants of the GAGA ZF derived from *Drosophila melanogaster* in HeLa cells and found that both the wild type and its deficient mutant were capable of translocating into the cytoplasm of HeLa cells without CPP.^56^ In this study, we used Sp1 ZF, a human transcription factor-derived protein consisting of three-finger units, as a new target protein.^58,59^ The Sp1 ZF consists of three Cys_2_His_2_-type ZF domains (1F1, 1F2, and 1F3) that link to form a tandem structure (Fig. 1). Furthermore, the protein can bind to a highly homologous DNA sequence (GGGCGGGG). Here, full-length Sp1 ZF (3F123) and ZFs with different numbers of finger domains (1F1, 1F2, 1F3, 2F12, and 2F23) were created (Fig. 1) to investigate the effects of the number of finger units, total cationic charge, and two-dimensional cation cluster structure formed *via* folding on the protein surface on the cell membrane permeability of ZFs.

## Results and discussion

### Observation of subcellular distribution of Sp1 ZFs using living HeLa cells

**Fig. 2:**
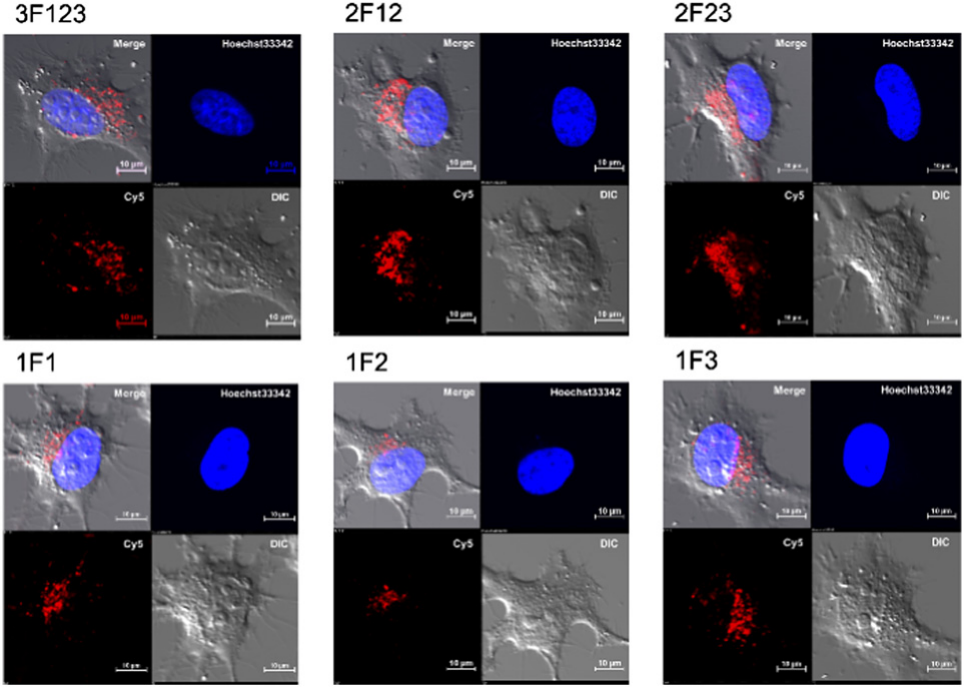
Cy5-labeled Sp1 ZFs (Sp1-3F123, 2F12, 2F23, 1F1, 1F2, and 1F3) transduced into HeLa cells. Peptide localization in the cells was observed using confocal microscopy: scale bar, 10 μm.

Membrane permeabilization experiments for each Cy5-labeled Sp1ZF were performed using living HeLa cells, and the localization of the peptides in cells was observed using confocal microscopy (Fig. 2). Since serum has previously been shown to significantly inhibit protein uptake into cells, cellular uptake experiments were performed using serum-free Opti-MEM to avoid non-specific interactions between ZF and serum proteins. In addition, cell fixation significantly impacts the observation of the subcellular localization of peptides using confocal microscopy.^60^ Therefore, living cells were used in our experiments. Hoechst 33342 was used to stain the nuclei, which presented a blue fluorescence (Fig. 2). Punctate red fluorescent signals were observed around the nucleus for all peptides, indicating that Sp1 ZFs had permeated the cell membrane and entered cytoplasm without the external CPP. Based on our previous observations regarding cell membrane permeabilization using GAGA ZF, we speculate that the punctate fluorescence signals may be due to the uptake of peptides *via* endocytosis during intracellular transfer and internalization into endosomes in the cytoplasm.^56^ To understand the mechanism of the intracellular delivery of Sp1 ZFs, we performed intracellular permeabilization experiments in Sp1-1F3 at 4 °C, as ATP-dependent endocytic processes halt at this temperature (Fig. S1, ESI†). The punctate fluorescence signals around the nucleus observed at 37 °C were not observed at 4 °C, indicating that the cell membrane permeation of Sp1 ZF did not occur directly through the cell membrane, but through an energy-dependent process such as endocytosis. Gaj *et al.* have examined endocytic pathways involved in the cellular uptake of ZFs, using Dynasore and Nystatin, which inhibit clathrin- and caveolin-dependent endocytosis, and Amiloride and Cytochalasin D, which inhibit macropinocytosis.^53^ Their results indicate that ZFs are mainly taken up into cells by macropinocytosis and less frequently by caveolin-dependent endocytosis. It is likely that Sp1 ZFs are taken up by a similar endocytosis pathway.

In addition, the cytotoxicity of ZF treatment for 2 and 12 hours was examined by WST-8 assays (Fig. S2, ESI†). As a result, no significant difference between ZF-treated and non-treated cells was observed at both time points, indicating no significant cytotoxicity of ZF treatment.

### Comparison of intracellular trafficking of Sp1 ZFs with various fingers

**Fig. 3:**
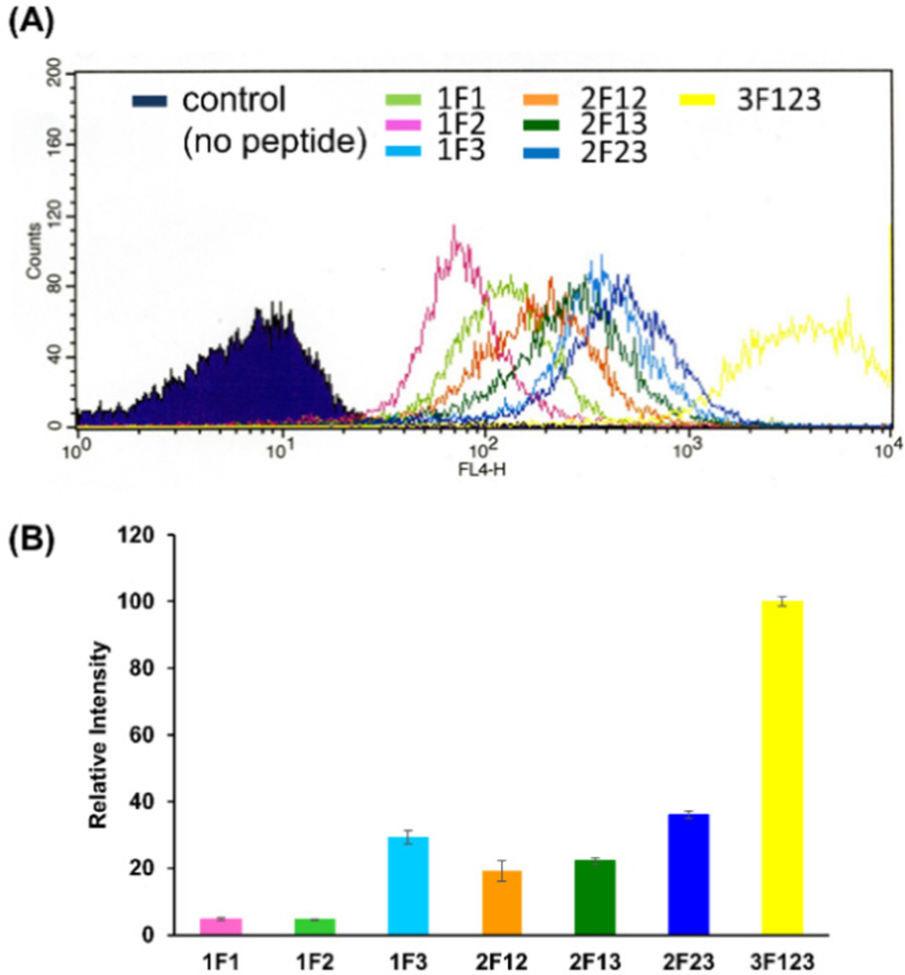
Flow cytometry analysis showing the amounts of internalized Cy5-labeled Sp1 zinc finger peptides (1F1, 1F2, 1F3, 2F12, 2F23, 2F13, and 3F123) into HeLa cells. (A) The normalized mean fluorescence intensity of Cy5-labeled Sp1 ZFs (Sp1-1F1, 1F2, 1F3, 2F12, 2F13, 2F23, and 3F123) was determined using flow cytometry (B).

We quantified the intracellular transport of each Sp1 ZF by determining the geometric mean of their fluorescence intensity in the intracellular membrane permeability experiment using flow cytometry and compared their cell permeabilities (Fig. 3). The extent of the cellular uptake of Sp1 ZFs increased in the order of 3F123 > 2F23 > 1F3 > 2F13 > 2F12 > 1F1 > 1F2, based on the number of finger domains, except for 1F3. Gaj *et al.* created tandem ZFs consisting of one to four identical single finger domains linked *via* a linker and examined their membrane permeability. The authors found that up to three finger domains had increased cellular uptake as the number of finger domains increased, suggesting that the effect of increased positive charge, rather than steric hindrance, due to the increased number of finger domains facilitated cellular uptake.^53^ We have previously shown that cellular uptake efficiency mainly depends on the magnitude of the net positive charge of the peptide.^56^ The theoretical value of the total charge of each Sp1 ZF was determined from the equation (*N*_Arg_ + *N*_Lys_) − (*N*_Glu_ + *N*_Asp_) (Table 1), where *N*_a.a._ is the total number of amino acids present in the primary sequence (Arg and Lys are positively charged, while Glu and Asp are negatively charged). As a result, the total cationic charge of each Sp1 ZF was as follows: 3F123 > 2F13 > 2F23 = 2F12 > 1F3 > 1F1 > 1F2. A comparison of the charge and cellular uptake revealed that the extent of intracellular translocation tended to increase with the total cationic charge of the protein, consistent with our previous findings and those of Gaj *et al.* and Liu *et al.*^52–54^ However, the single-finger domain, 1F3, was an exception; the cellular uptake efficiency of 1F3 (+7) was unexpectedly higher than that of other single fingers 1F1 (+5) and 1F2 (+4). In addition, 1F3 (+7) showed significantly higher cellular uptake than two-finger domains 2F12 (+10) and 2F13 (+12). These results suggest that the cellular uptake efficiency of ZFs was not only controlled by the total number of positive charges. Therefore, we focused on the effect of the folding structure of the finger domain.

**Table 1:** Total number of cationic and anionic amino acid residues in Sp1 peptides and their total net charge

	Total amino acid residues	Arg	Lys	Glu	Asp	Total net charge
1F1	35	2	5	1	1	+5
1F2	30	5	2	2	2	+4
1F3	29	2	7	1	1	+7
2F12	67	7	8	3	3	+10
2F13	65	4	12	2	2	+12
2F23	63	7	9	4	4	+10
3F123	95	9	14	4	4	+16

### Effect of the folded structure of ZF domains on intracellular membrane permeability

Previous reports have suggested that a well-dispersed arrangement of arginine on the surface of a folded structure is important for the efficient cellular uptake of proteins.^50^ As Sp1 ZF is a DNA-binding domain, its surface predominantly harbors cationic amino acids such as arginine and lysine, resulting in a positively charged surface. We hypothesized that the two-dimensional cation clusters on the surface of Sp1 ZF formed after folding may play an essential role in the high intracellular transport of 1F3 peptides. Therefore, we investigated the relationship between the formation of two-dimensional cation cluster structures and intracellular trafficking. For this purpose, we constructed an experimental system that could control the secondary and tertiary structures of target proteins under mild conditions and enabled cell culture without the use of cytotoxic denaturing agents such as guanidinium or urea and/or without extensive mutations. In the case of ZFs, the folded structure can be easily controlled by replacing the coordinating amino acid in the zinc ion coordination site with a non-coordinating amino acid, as the folded structure cannot form without Zn^2+^ coordination. This allowed us to directly evaluate the effect of the two-dimensional cation cluster structure induced during folding on membrane permeability. Another advantage of this method is that it allowed us to create random structural states under conditions where the total charge of the ZFs remains unchanged. This showed that ZFs were suitable model proteins for the present study. Therefore, we prepared mutants (1F1-Ala, 1F2-Ala, and 1F3-Ala) wherein the two Cys residues involved in the Zn^2+^ coordination of each finger domain were replaced with non-coordinating Ala residues. Normally, under physiological conditions, the SH groups of the two Cys residues of ZFs are coordinated with Zn^2+^ in the form of thiolate anions; hence, only the two Cys residues contribute −2 charge to the total charge of ZFs. However, in the C_2_H_2_-type ZF, the net charge at the coordination site is considered to be zero, as the two anionic Cys residues and the two neutral His residues form a coordinated structure with divalent Zn^2+^. Furthermore, as Zn^2+^ is not coordinated to ZFs in Cys to Ala point mutants, the charge of Zn^2+^ does not have to be considered in the Ala mutant, and this point mutation is not expected to alter the total charge of ZFs.

First, we analyzed the folding properties of each Ala mutant using CD measurements, as the CD signature of Sp1 ZF is known (Fig. S3, ESI†). In general, the apo forms of wild-type ZFs present a minimum value at approximately 200 nm in the CD spectrum, which indicates that most apo forms of ZFs have a random structure in aqueous solutions.^58,59^ However, in the CD spectra of Zn^2+^-coordinated wild-type ZFs, the signals derived from the ββα structure were observed at approximately 208 and 222 nm.^58,59^ In the absence of Zn^2+^, all Sp1 Ala mutants showed negative CD bands at approximately 200 nm, suggesting that most of the peptides in the apo form were in the random coil state. Next, when Zn^2+^ was added to 1F1-Ala and 1F2-Ala, the CD spectra of both were similar to those of the respective apo forms. A slight change in the CD spectrum of 1F3 was observed at around 220 nm, which differed completely from the CD spectrum of the wild type with Zn^2+^. We observed that unlike the CD spectrum derived from the typical ββα structure of the wild type, that of the Ala mutant in the presence of Zn^2+^ presented a minimum value at around 200 nm, indicating that the Ala mutants mostly have random structures in an aqueous solution. Thus, we succeeded in disrupting only the structure of Sp1 ZF without changing its total charge.

**Fig. 4:**
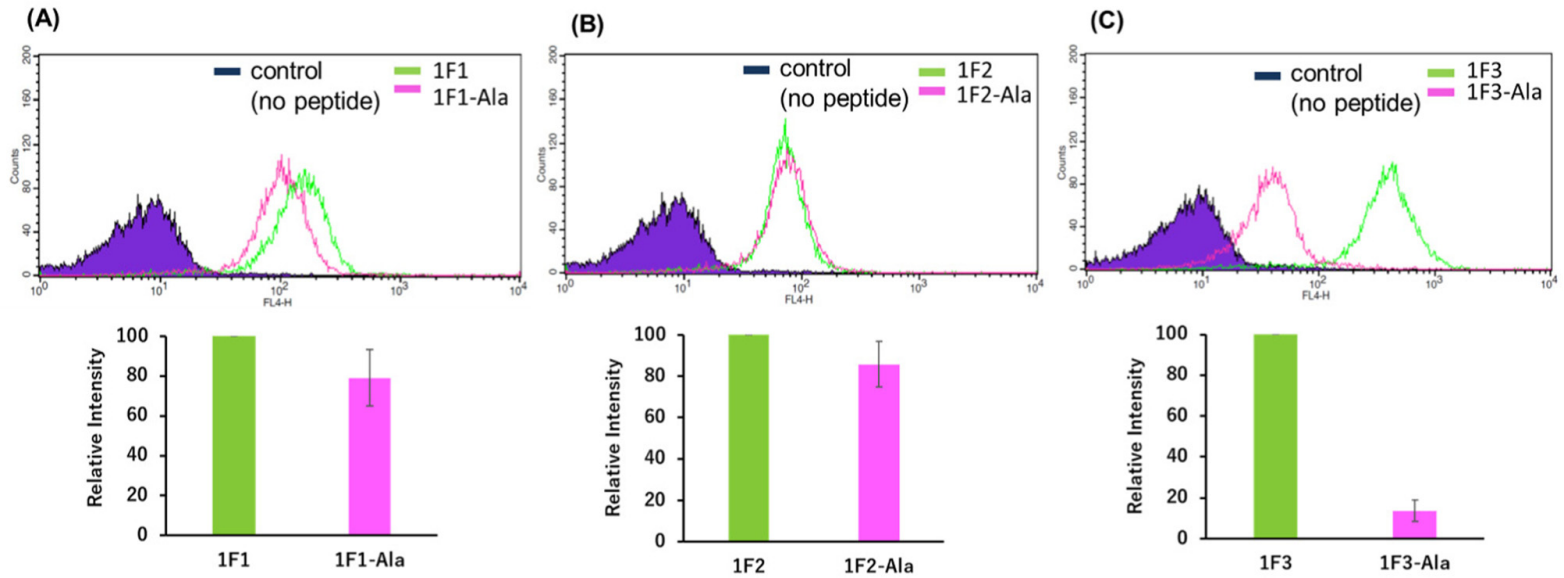
Flow cytometry analysis showing the amount of internalized Cy5-labeled Sp1 zinc finger peptides ((A): 1F1 and 1F1-Ala, (B): 1F2 and 1F2-Ala, and (C): 1F3 and 1F3-Ala) into HeLa cells. The geometric mean of the fluorescence intensity of Cy5-labeled Sp1 ZFs (1F1 and 1F1-Ala (A), 1F2 and 1F2-Ala (B), and 1F3 and 1F3-Ala (C)) in HeLa cells was determined using flow cytometry.

Next, we examined the relationship between the two-dimensional cationic clusters and the ability of Sp1 ZFs to enter cells by comparing the uptake of each structured ZF and their unstructured Ala mutants by HeLa cells using confocal microscopy and flow cytometry. Confocal microscopy revealed that similar to that observed for the wild type, each Ala mutant showed punctate red fluorescence around the nucleus, indicating that they had penetrated the plasma membrane and entered the cytoplasm to localize around the nucleus (Fig. S4). Flow cytometry showed that the intracellular uptake of 1F3-Ala decreased by approximately 80% compared to that of the 1F3 wild type, indicating a marked difference in the amount of intracellular transfer (Fig. 4). In the case of 1F1 and 1F2, the cellular uptake of the Ala mutant was lower than that of the wild type, although not to the extent observed for 1F3, as the uptake of the wild type was inherently low (Fig. 4a and b). Interestingly, the intracellular trafficking of the Ala mutants was similar, indicating that the loss of the folded structure of each finger domain diminished the contribution of the total cationic charge to membrane permeability. In short, although the total cationic charge of ZF remained unaltered, the loss of conformation decreased the efficiency of translocation across the cell membrane. Therefore, in addition to the total cationic charge of the protein, the two-dimensional cation cluster structure formed on its surface is one of the most important factors responsible for its ability to permeate the cell membrane.

### Effect of cationic amino acid clustering sites on the primary sequence of Sp1-1F3 on cell membrane permeability

**Fig. 5:**
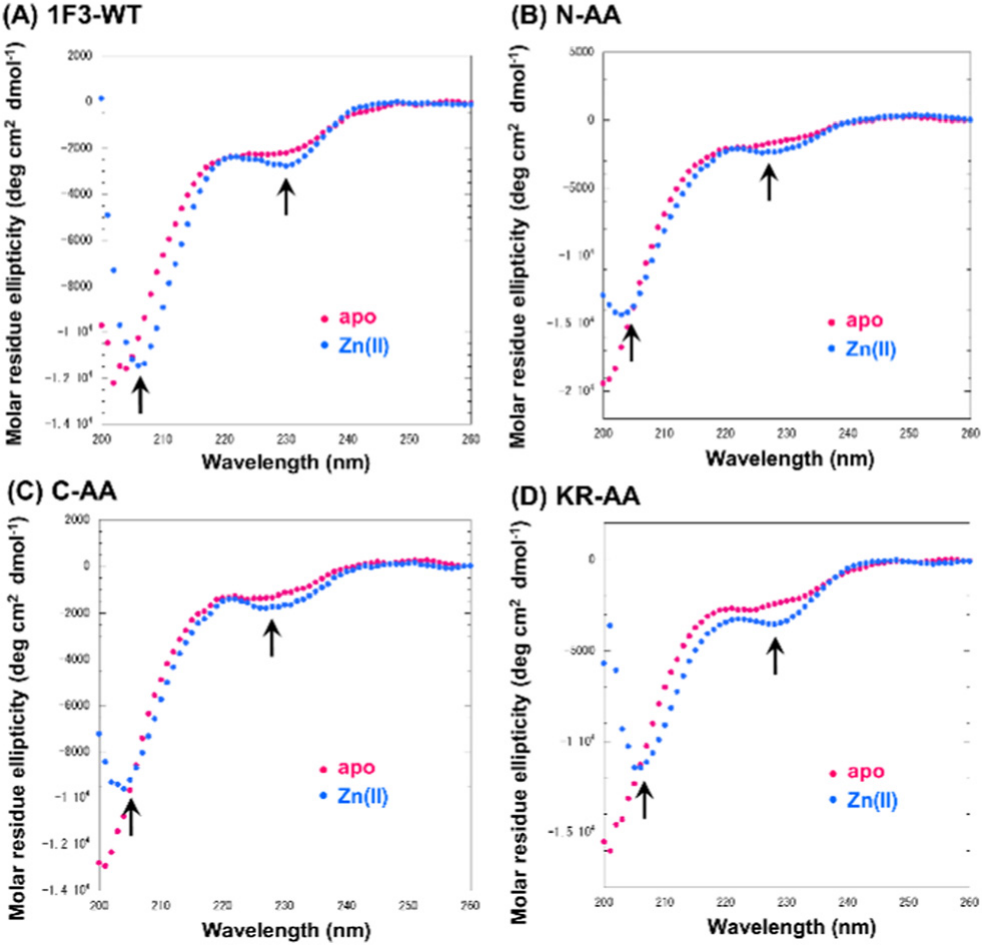
Circular dichroism spectra of Sp1-1F3-WT (A), N-AA (B), C-AA (C), and KR-AA (D) (peptide concentration; 20 μM) in the absence (apo) and presence of 1.5 eq. metal ions Zn^2+^ in 10 mM Tris-HCl buffer (pH 7.5) containing 50 mM NaCl at 20 °C.

Next, we focused on the basic amino acids in the primary sequence of 1F3 involved in the formation of the two-dimensional cation cluster structure. We selected three consecutive cluster sequence sites of two basic amino acids in the primary sequence of 1F3 and created mutants wherein an Ala residue replaced each amino acid. The N-AA, C-AA, and KR-AA were mutants in which the N-terminal KK and C-terminal KK derived from the conserved linker region, and the KR sequences located in the middle of the sequence derived from the finger domain, were replaced by Ala, respectively (Fig. 1). Michael *et al.* synthesized artificial peptides in which all, except for the seven most conserved residues characteristic of the TFIIIA-type ZF domain, were replaced with alanine and examined their metal binding and structural properties.^61^ The authors showed that the Ala mutant peptide complexed with Zn^2+^ to form a three-dimensional structure very similar to that of the wild type. This indicates that Zn^2+^ binding and folding ability is retained even when amino acids other than the conserved sequence of ZFs are mutated. Based on this, we examined the CD spectra of the three Ala mutants with respect to structure formation. We found that these mutants form a random structure in the apo form, similar to that observed in wild-type 1F3, and, in the presence of Zn^2+^, form the typical ββα folding structure observed in ZFs, as shown by minimum values of 208 and 222 nm in the CD spectrum (indicated by black arrows; Fig. 5). This result is similar to that reported by Michael *et al.*, indicating that the folded structure is not lost by these mutations.^61^

**Fig. 6:**
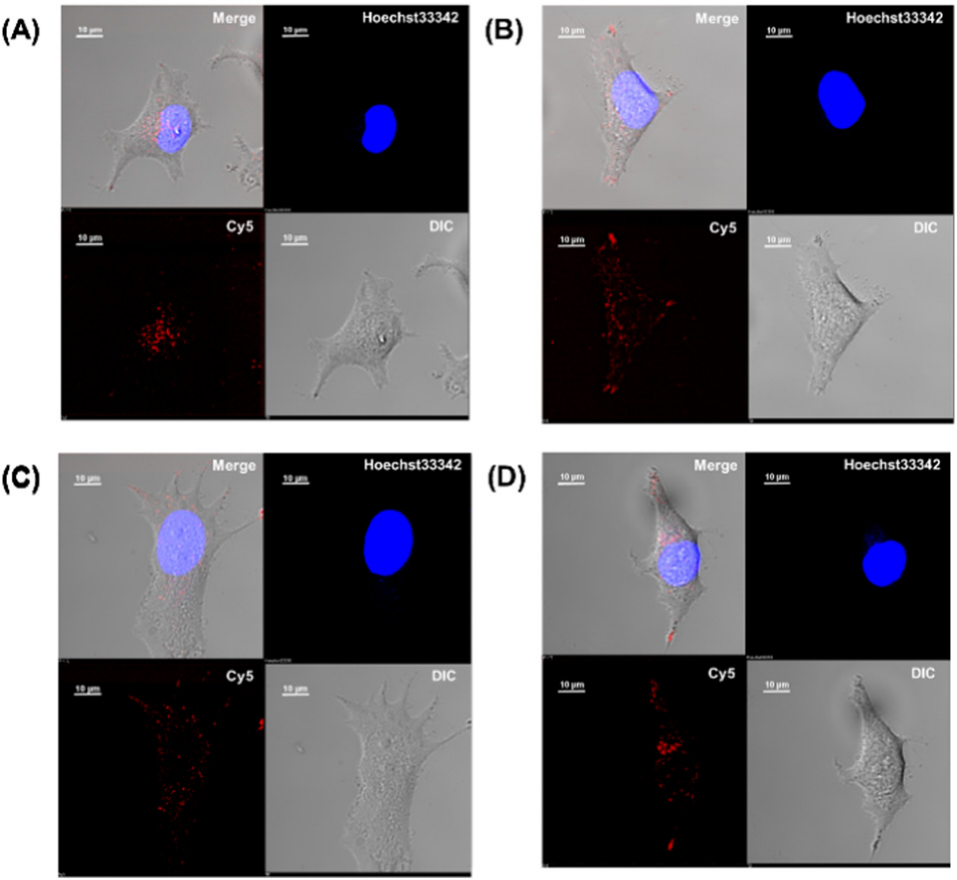
Cy5-labeled Sp1 ZFs (Sp1-1F3-WT (A), N-AA (B), C-AA (C), and KR-AA(D)) were transduced into HeLa cells. Peptide localization in the cells was observed using confocal microscopy: scale bar, 10 μm.

**Fig. 7:**
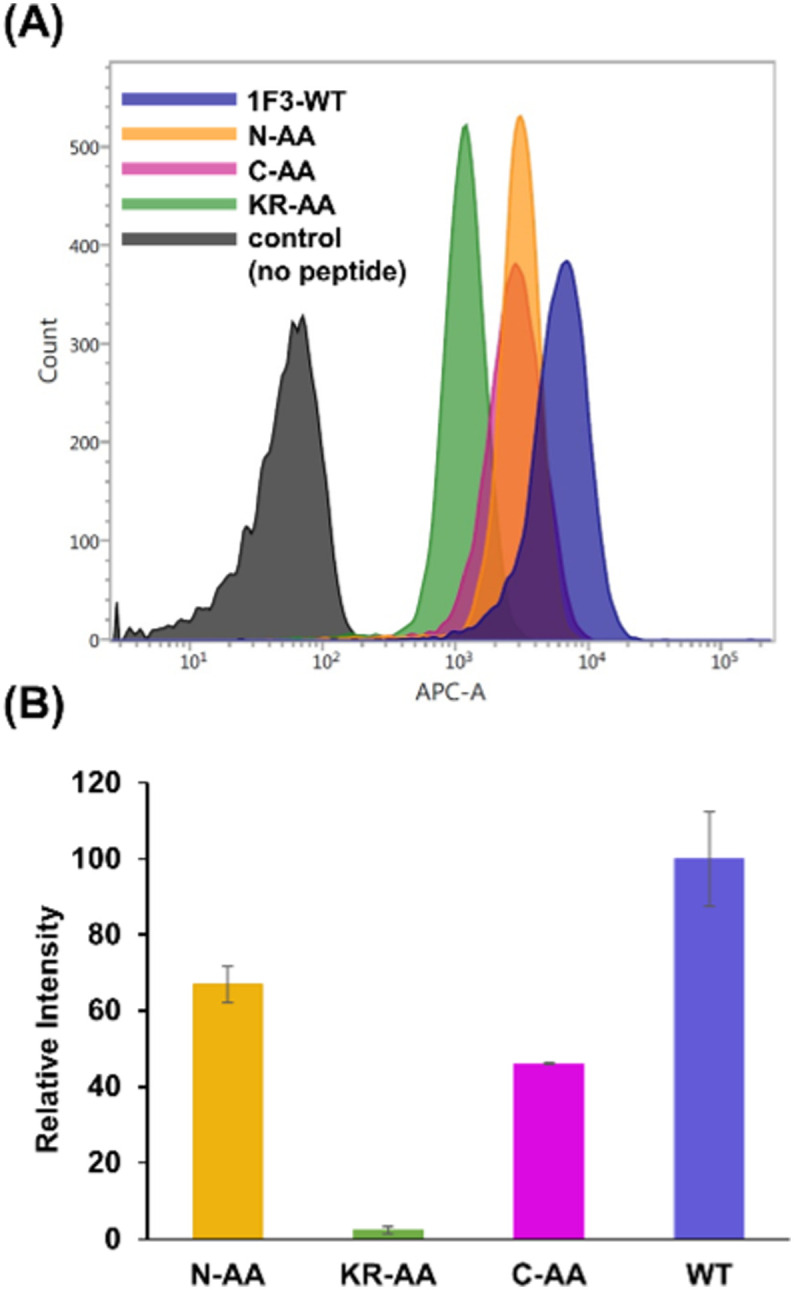
Flow cytometry analysis showing the amount of internalized Cy5-labeled Sp1 zinc finger peptides (Sp1-1F3-WT, N-AA, C-AA, and KR-AA) into HeLa cells. (A) The normalized mean fluorescence intensity of Cy5-labeled Sp1 ZFs (Sp1-1F3-WT, N-AA, C-AA, and KR-AA) in HeLa cells was determined using flow cytometry (B).

The intracellular translocation ability of mutants was examined using confocal microscopy and flow cytometry. The results of confocal microscopy showed punctate fluorescent signals around the nucleus, confirming that each mutant migrated into the cell and localized around the nucleus (Fig. 6). This suggests that the three Ala mutants were incorporated by the cell *via* the same mechanism as the wild type. Furthermore, the quantitative intracellular membrane permeability of each mutant was examined using flow cytometry (Fig. 7). The results showed that the intracellular transfer of mutants was lower than that of the wild type. However, although the total cation charge of each Ala mutant was lower by +2 than that of the wild-type, the Ala mutants differed considerably in the extent of intracellular transfer. In particular, the KR-AA mutant was transferred the least, indicating that the cation cluster sequence located in the finger domain significantly affected intracellular transferability.

**Fig. 8:**
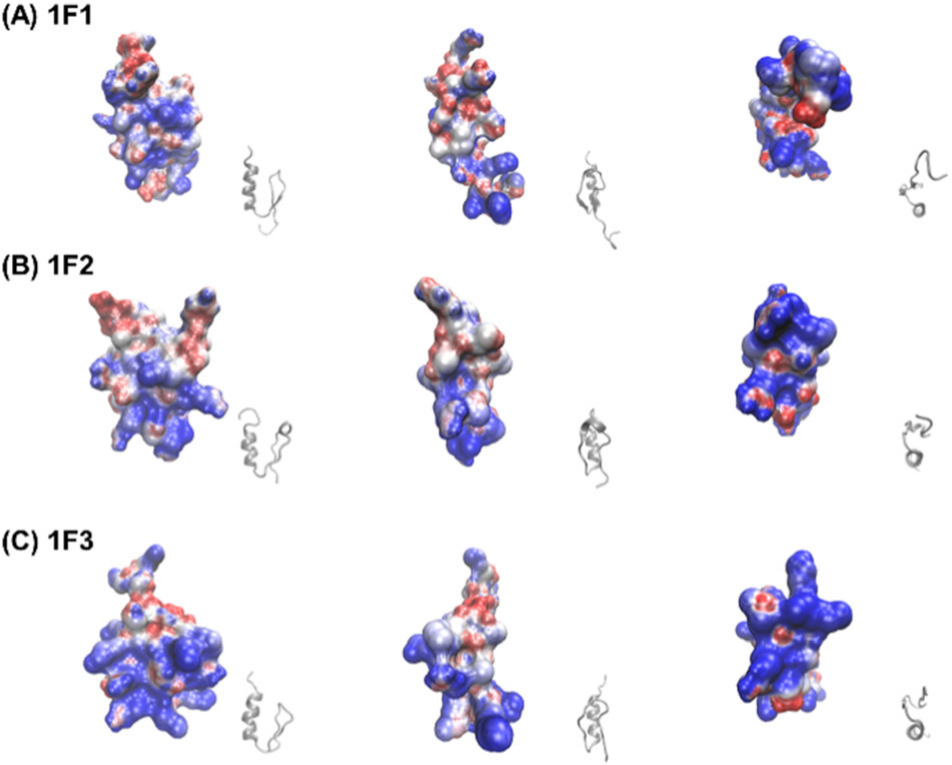
Space-filling model depicting the charge distribution states on the surface of Sp1-1F1 (A), 1F2 (B), and 1F3 (C) (blue: cations, red: anions, white: neutrals) and the corresponding ZF framework structure (gray).

Furthermore, the clustering region of cationic amino acids on the primary sequence is important for forming a two-dimensional cation cluster on the domain surface *via* folding. To study the structural information of the two-dimensional cation clusters formed on the domain surface, we calculated the surface charge of 1F1, 1F2, and 1F3, represented by space-weighted models built based on the structural information obtained from NMR structural analysis.^62^ Protein structures and models were visualized using VMD.^63^ The surface charge is illustrated in red for the negatively charged area, in blue for the positively charged area, and in white for the neutral area, respectively (Fig. 8 and Movies S1–S3, ESI†). In the case of 1F1, positive (blue) and negative (red) charges were evenly distributed over the entire surface, indicating a low pronounced localization of positive charges (Fig. 8a and Movie S1, ESI†), while the positive charge was localized on the surface of 1F2. However, compared to that in 1F1, the cluster sites of the positive charge in 1F2 were located in an inner cleft (Fig. 8b and Movie S2, ESI†). On the contrary, compared to that observed in other finger domains, a positive charge accumulation region (blue region) was detected on one side of the 1F3 surface, which may be responsible for the high intracellular membrane permeability of 1F3 (Fig. 8C and Movie S3, ESI†).

**Fig. 9:**
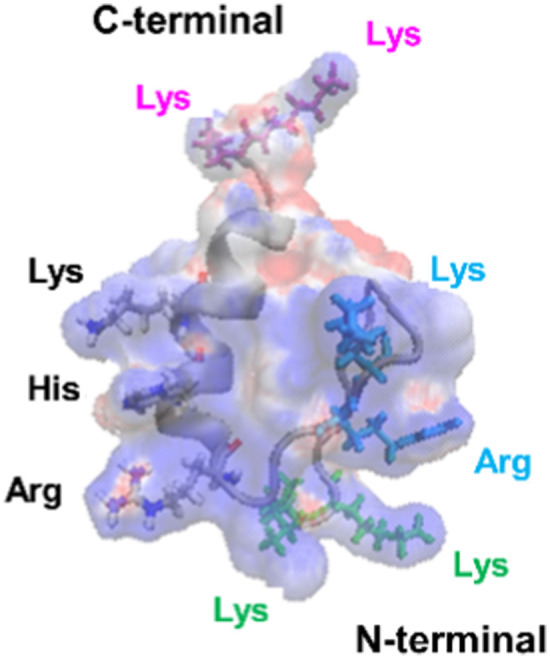
Superimposed structure of basic amino acid residues involved in the formation of cation clusters on the charge distribution diagram of 1F3 shown in the left panel of Fig. 8C. The N-terminal KK sequence, C-terminal KK sequence, and KR sequence of 1F3 are represented in green, pink, and blue, respectively.

The mechanism underlying the high mobility of 1F3 was further investigated using the superimposed image of the positively charged part of the cation cluster shown in the left panel of Fig. 8c and the basic amino acid residues involved in its formation (Fig. 9). The results clearly showed that the cation clusters efficiently are formed by the proper arrangement of several basic amino acids on one side of the 1F3 surface. Furthermore, the cation cluster regions can be broadly classified into three types: the two terminal portions consisting of N-KK and C-KK sequences, the interior of the finger domain consisting of the KR sequence, and the Lys, His, and Arg residues located on the opposite side. In all mutants of N-AA, C-AA, and KR-AA, the amount of intracellular transfer was lower than that of the wild type. This may be because the substitution of a basic amino acid with an Ala residue disrupted a part of the well-formed two-dimensional cation cluster, in addition to reducing the positive charge. Furthermore, among the three mutants, the most striking decrease in cell membrane permeability was observed for the KR-AA mutant.

We hypothesized that one reason for the difference in membrane permeability for the three mutants may be due to differences in the structural fluctuations in the three regions of the cation cluster described above. The cationic portions of both the N-terminal and C-terminal KK sequences were exposed on the surface, which may be advantageous for cell entry. However, an NMR analysis of 1F3 revealed that these areas fluctuate substantially in aqueous solutions,^62^ which may prevent their close interaction with the cell membrane surface. In contrast, the KR sequence, together with other basic amino acids, forms a two-dimensional surface-exposed cation cluster at the center of the finger domain surface; furthermore, the NMR results indicated that the fluctuations in this area are small and that it forms a relatively rigid structure. These results suggest that the two-dimensional cation clusters, which form over a wide area on the surface of the protein and have few fluctuations, play an important role in the permeation of proteins and peptides through the cell membrane. Moreover, a comparison between the cell membrane permeability of oligopeptides with equal numbers of Arg and Lys residues has shown that the former often exhibit a several times higher intracellular translocation than the latter, suggesting that the Arg residue is more important for cellular uptake.^64^ Thus, it may also be necessary to consider that the large reduction in cellular uptake observed in the KR-AA mutant is due to Ala substitution of Arg residues in the KR sequence, unlike for the other two mutants. Although the present results are interesting, it is necessary to study other proteins, including ZFs, to show more generality.

## Conclusions

In this study, we investigated the effects of the number of finger domains of the Sp1 ZF target protein and the two-dimensional cation cluster structure consisting of basic amino acids on membrane permeability using confocal microscopy and flow cytometry measurements. We observed that all Sp1 ZFs used in this study were taken up by the cell *via* endocytosis and were distributed around the cell nucleus in the endosomal state. Furthermore, the amount of intracellular trafficking increased with the number of finger domains, *i.e.*, the total positive charge of ZFs. However, the intracellular translocation of 1F3 and finger domains containing 1F3 deviated from this observation, suggesting that factors other than total cation charge affected intracellular translocation. We also examined the membrane permeability of the 1F3 mutant. The two Cys residues involved in Zn^2+^ coordination were mutated to Ala residues, which did not change the total cationic charge. The amount of intracellular translocation markedly decreased compared to that of the wild type, indicating that a folded structure is important for intracellular translocation efficiency. The spatial modeling of 1F3 indicated that the less fluctuating, surface-exposed, and two-dimensional cation cluster structures formed on the surface of the finger domain portion of 1F3 played an important role in the intracellular translocation ability of 1F3. The interaction of basic peptides enriched in Arg and Lys residues with proteoglycans on the cell surface was important for the permeability of the cell membrane. Therefore, it is expected that the formation of a two-dimensional cation cluster structure with an appropriate spatial arrangement and less fluctuation on the surface of the peptide/protein during folding can lead to more efficient interaction with proteoglycans, which are also present on the surface of the cell membrane, activating the signal for endocytosis and increasing the efficiency of the intracellular transfer of peptides and proteins. Therefore, GFP with its rigid backbone, which has been used for preparing supercharged proteins, is considered to be one of the best templates for the formation of two-dimensional cation clusters on protein surfaces. In the future, natural proteins that can act as templates for new carriers could be identified based on this perspective. The ZF is also one of the best templates for natural carriers, and it is expected that other ZFs with higher membrane permeability will be investigated in the future. Alternatively, artificial proteins with efficient membrane permeability could be created by redesigning natural ZFs. Meanwhile, since ZFs are internalized into endosomes within the cell, another question remains as to how these proteins escape from endosomes and function in the cytoplasm or nucleus. This point needs to be further investigated for the application of ZFs as practical transport vectors. However, the findings of this study not only provide fundamental insights regarding the mechanism of cell membrane permeability but will also be useful in designing vectors for transferring target substances into cells more efficiently.

## Data availability statement

The datasets supporting this article have been uploaded as part of the ESI.†

## Author contributions

S. N., M. H., M. I. and Y. K. N. designed the project, conducted molecular biology and biochemical experiments, analysed data, and contributed to the writing of the manuscript. M. K., C. K., Y. K., and K. K. conducted molecular biology and biochemical experiments and analysed data. T. M., N. K. focused on confocal microscopy and flow cytometry experiments and provided discussion and support in the preparation of the paper. H. K., M. M., N. S., and Y. S. helped design the project and to write the manuscript. All authors contributed to the proofreading of the manuscript.

## Conflicts of interest

There are no conflicts to declare.

## Supplementary Material

CB-003-D2CB00098A-s001

CB-003-D2CB00098A-s002

CB-003-D2CB00098A-s003

CB-003-D2CB00098A-s004
