# Supplementary material for: Importance of two-dimensional cation clusters induced by protein folding in intrinsic intracellular membrane permeability
Source: RSC Chem Biol. 2022 Jul 13;3(8):1076–84. doi: 10.1039/d2cb00098a (PMC9347356; doi:10.1039/d2cb00098a)
Supplement: CB-003-D2CB00098A-s001 [file CB-003-D2CB00098A-s001.pdf]

Electronic Supplementary Material (ESI) for RSC Chemical Biology.
This journal is © The Royal Society of Chemistry 2022

Supporting Information

Importance of two-dimensional cation clusters induced by protein folding in intrinsic intracellular membrane permeability

Shigeru Negia*, Mami Hamori, Yuka Kawahara-Nakagawa, Miki Imanishi, Miku Kurehara, Chieri Kitada, Yuri Kawahito, Kanae Kishi, Takayuki Manabe, Nobuyuki Kawamura, Hiroaki Kitagishi, Masato Mashimo, Nobuhito Shibata and Yukio Sugiura

Contents

1. Preparation and Cy5 labeling of Sp1 ZFs
2. Circular dichroism (CD) measurements
3. Cell culture
4. Confocal microscopy
5. Flow cytometry

Experimental Procedures

Preparation and Cy5 labeling of Sp1 ZFs

The DNA fragment encoding the Sp1 ZF containing three-finger domains (3F123) was obtained from Life Technologies (Carlsbad, CA, USA) and used as an insert to construct the protein expression vector. The insert DNA was cleaved using NdeI and EcoRI restriction enzymes and inserted into the multi cloning site of the pET-17b vector (Novagen, Madison, WI, USA). The sequence of the constructed expression vector was confirmed using a 3130 Genetic Analyzer (Thermo Fisher Scientific, Waltham, MA, USA). Rosetta^{TM2} (DE3) pLysS Escherichia coli competent cells (Novegen, Madison, WI, USA) were grown at 37 °C in the presence of 0.1 M isopropyl-β-D-thiogalactopyranoside (IPTG) and 0.1 M $ZnCl_2$ until an optical density at 600 nm (OD_{600}) of 0.6 was reached. Then, the culture was incubated for an additional 12 h at 20 °C in the presence of 0.1 M IPTG and 0.1 M $ZnCl_2$ to obtain soluble Sp1 ZF. The overexpressed proteins were purified using cation-exchange chromatography on a Bio-Logic LP system (Bio-Rad, Hercules, CA, USA) and desalted using a Bio-Scale^{TM} Mini Macro-Prep High S (Bio-Rad) cartridge. Final purification was performed using a Cosmosil 5C18-ARII (10 x 250 nm; Nacalai Tesque, Inc., Kyoto, JPN) reverse-phase high-performance liquid chromatography (RP-HPLC) column. The purity of the purified protein was confirmed using sodium dodecyl sulfate polyacrylamide gel electrophoresis (SDS PAGE) (data not shown) and matrix-assisted laser desorption ionization time-of-flight mass spectroscopy (MALDI-TOF-MS). The ultrafleXtreme and its supplied software (Bruker, Billerica, MA, USA) were used for MALDI-TOF-MS. Two-finger domains (2F12 and 2F23) of Sp1 ZF were also prepared using the same method.

Sp1 single finger domains (1F1, 1F2, 1F3) and Sp1 mutant peptides (1F1-Ala, 1F1-AE, 1F2-Ala, 1F3-Ala, 1F3-RAR) (Figure 1) were purified using the TGS-RAM resin (Shimadzu Corporation, Kyoto, JPN) and a standard Fmoc solid-phase method (2-(1H-benzotriazole-1-yl)-1, 1, 3, 3-tetramethyluronium hexa-fluorophosphate (HBTU)/1-hydroxy benzotriazole (HOBT) as the coupling reagent) with a PSSM-8 synthesizer (Shimadzu Corporation, Kyoto, JPN). The synthesized peptides were removed from the resin by incubating with 86% trifluoroacetic acid, 2.5% water, 5% 1, 2-ethanedithiol, 5% thioanisole, and 1.5% triethylsilane for 4 h. The crude peptides were then precipitated in ice-cold ether, separated via centrifugation at 8000 x g for 5 min at 4 °C, washed thrice with cold diethyl ether, dissolved in water, and lyophilized. The peptide was purified using a Cosmosil 5C18-ARII (10 x 250 nm; Nacalai Tesque, Inc., Kyoto, JPN). The identity of the products was confirmed using MALDI-TOF-MS. To fluorescently label Sp1 ZF, a Lys-Cys-Lys sequence was introduced at the C-terminal side. Each peptide was fluorescently labeled by adding 3, 4, and 6 equivalents of Cy5 (GE Healthcare, Little Chalfont UK) dissolved in 0.1 M HEPES (pH 8.0) to obtain 1F, 2F, and 3F, respectively, and reacted for 18 h at 25 °C, followed by purification using Cosmosil 5C18-ARII. The Cy5-labeled peptides were confirmed using MALDI-TOF-MS. The MALDI-TOF-MS data are summarized in Table S1.

Circular dichroism (CD) measurements

CD was measured using a JASCO J-720 spectropolarimeter (Jasco, Tokyo, JPN). The spectra were recorded in the range of 195 - 260 nm in the continuous mode with a bandwidth of 1 mm, response time of 1 s, and scan speed of 20 nm/min. Each spectrum was represented by the average of 20 scans at 20 °C in Tris-HCl buffer (50 mM, pH 7.5) containing NaCl (50 mM) and tris(2-carboxyethyl) phosphine (TCEP) (25 μM) in a capped 0.1 cm path length cell under a nitrogen atmosphere.

Cell culture

HeLa cells were purchased from the Riken BRC Cell Bank (Ibaraki, JPN) and cultured in α-minimal essential medium (MEM) supplemented with 10% heat-inactivated fetal bovine serum (Gibco, Grand Island, NY, USA) and MEM non-essential amino acids in T75-flasks at 37 °C in a 5% CO_2 incubator to approximately 70% confluence. The cells were sub-cultured every 3 - 4 days.

Confocal microscopy

HeLa cells (4 x 10^4 cells/mL) were plated on 35 mm glass-bottomed dishes (Iwaki, Tokyo, JPN) and cultured in a growth medium for 24 h at 37 °C in a 5% CO_2 incubator. The nuclei of the living HeLa cells were labeled with Hoechst 33342 by adding Cellstain® (Dojindo, Kumamoto, JPN) to the culture medium. The concentrations of Cy5-labeled peptide stock solutions were estimated using a spectrophotometer (absorbance of Cy5 at 650 nm; ε = 250,000, where ε is the extinction coefficient (M^{-1} cm^{-1})). The peptide stock solutions contained 1.5 equivalents of Zn(II) per one finger and 50 μM TCEP. The medium was replaced after 24 h with fresh Opti-MEM reduced serum medium (Thermo Fisher Scientific), and the cells were incubated for 2 h at 37 °C in the presence of 5% CO_2. The treated cells were washed five times with phosphate buffered saline (PBS) and the medium was replaced with fresh growth medium. The distribution of the Cy5-labeled peptides was analyzed using a confocal scanning laser microscope, A1R HD25 (Nikon Corp., Tokyo, JPN).

Flow cytometry

HeLa cells (2 x 10^5 cells/mL) were plated on 60 mm dishes (Iwaki, Tokyo, JPN) containing growth medium for 24 h at 37 °C in a 5% CO_2 incubator. The concentrations of the Cy5-labeled peptide stock solutions were spectrophotometrically estimated (absorbance of Cy5 at 650 nm; ε = 250,000, where ε is the extinction coefficient (M^{-1} cm^{-1})). The peptide stock solutions contained 1.5 equivalents of Zn(II) per one finger and 50 μM TCEP. The medium was replaced after 24 h with fresh Opti-MEM reduced serum medium (Thermo Fisher Scientific), and the cells were incubated for 30 min at 37 °C in the presence of 5% CO_2. The treated cells were washed five times with PBS and treated with 0.25% trypsin-EDTA (500 μL) for 5 min at 37 °C. The dissociated cells were suspended in PBS (2 mL) and separated via centrifugation at 200 x g for 10 min at 25 °C. Then, PBS (2 mL) was added to the treated cells from which the supernatant was removed. The fluorescence of the solution was analyzed on a FACSCalibur (BD Biosciences, Franklin Lakes, NJ, USA) flow cytometer and CELL SORTER SH800Z (SONY Corporation, Tokyo, JPN) using 488 nm excitation and 653 - 669 nm emission filters.

Results and Discussion

Table S1. Calculated and measured molecular weights of Sp1 zinc finger and Cy5-labeled peptides.

Peptide	Calculated	Measured
3F123	11573.75 (+Cy5 12351.75)	11572.349 (+Cy5 12348.163)
2F12	8323.53 (+Cy5 9101.53)	8321.989 (+Cy5 9101.990)
2F13	7876.18 (+Cy5 8654.18)	7877.206 (+Cy5 8655.402)
2F23	7871.04 (+Cy5 8643.90)	7742.192 (+Cy5 8522.825)
1F1	4366.13 (+Cy5 5144.13)	4367.424 (+Cy5 5145.429)
1F1-Ala	4302.00 (+Cy5 5080.00)	4302.626 (+Cy5 5082.210)
1F2	4073.73 (+Cy5 4850.73)	4074.086 (+Cy5 4852.685)
1F2-Ala	4009.53 (+Cy5 4787.83)	4010.431 (+Cy5 4789.592)
1F3	4045.89 (+Cy5 4823.89)	4044.421 (+Cy5 4822.824)
1F3-Ala	3981.76 (+Cy5 4759.76)	3981.655 (+Cy5 4760.655)
1F3-N-AA	3931.70 (+Cy5 4709.70)	3934.015 (+Cy5 4713.452)
1F3-KR-AA	3903.69 (+Cy5 4681.69)	3905.819 (+Cy5 4684.940)
1F3-C-AA	3931.70 (+Cy5 4709.70)	3932.489 (+Cy5 4713.937)

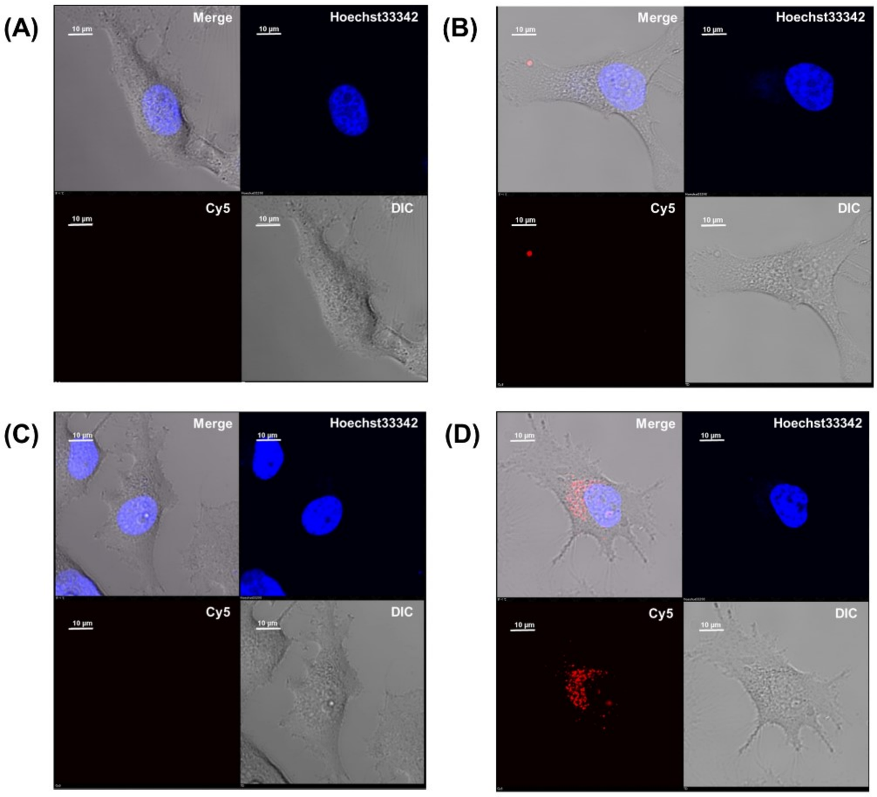

Figure S1. Internalization of control into HeLa cell at 4°C (A) or 37°C (C). Internalization of Cy5-labeled Sp1 1F3 into HeLa cell at 4°C (B) or 37°C (D). The localization of the peptides in the cells was observed by confocal microscopy (x20 objectivelens). ScaleBar, 10 μm.

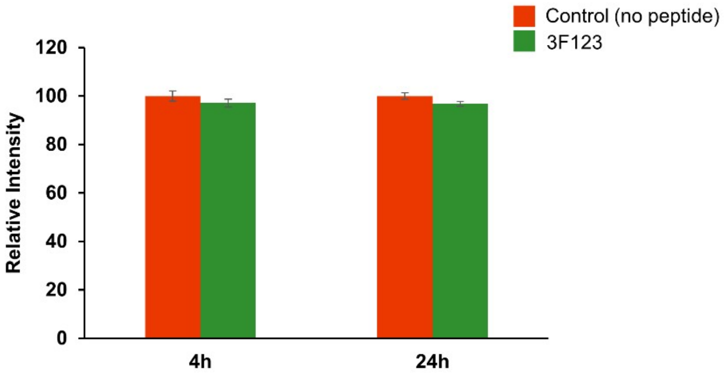

Figure S2. Cytotoxicity evaluation of Sp1F123 on HeLa cells. Cytotoxicity was evaluated by WST-8 assay. After 4 and 12 hours of incubation, respectively, those media were replaced and subjected to WST-8 assay.

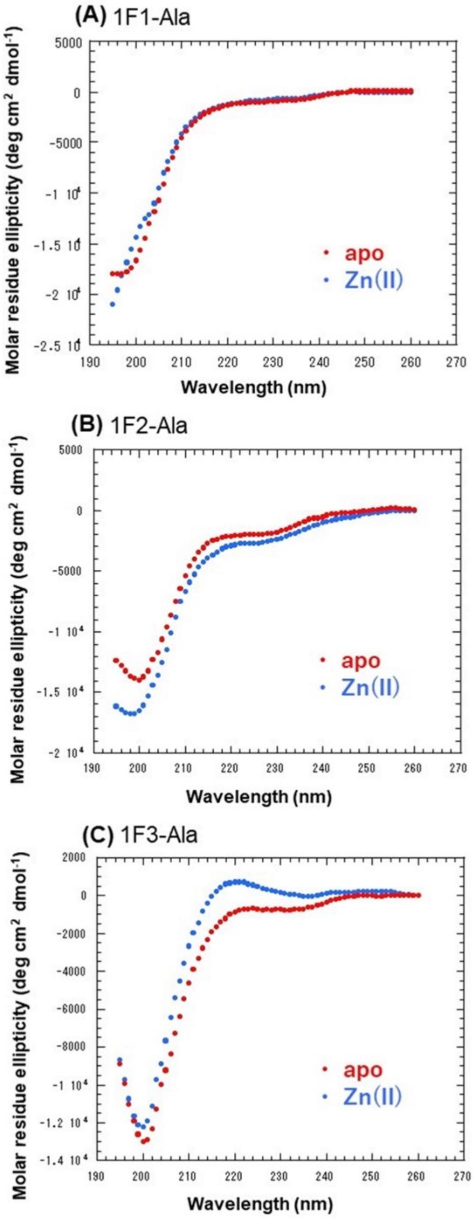

Figure S3. CD spectra of Sp1-1F1-Ala (A), 1F2-Ala (B) and 1F3-Ala (C) (peptide concentration; 20 mM) in the absence (apo) and presence of 1.5 eq. metal ions (Zn(II)) in 10 mM Tris-HCl buffer (pH 7.5) containing 50 mM NaCl at 20°C.

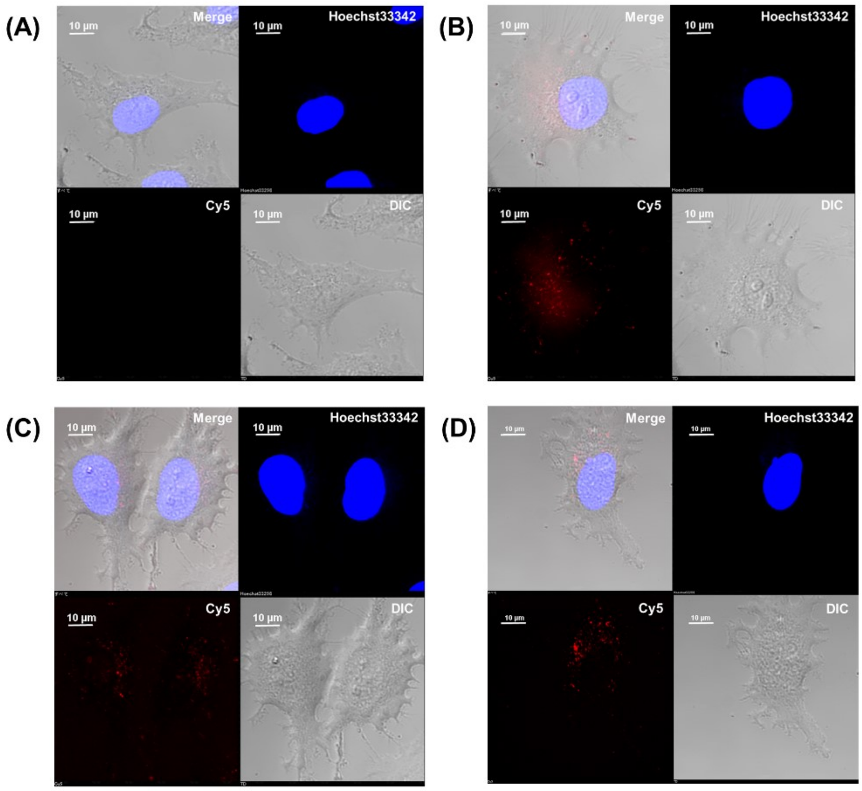

Figure S4. Cy5-labeled Sp1 zinc finger peptides (control (A), 1F1-Ala (B), 1F2-Ala (C), and 1F3-Ala (D)) transduced into the HeLa cells. The localization of the peptides in the cells was observed by confocal microscopy (x20 objectivelens). ScaleBar, 10 μm.
